# Electrical Percolation Threshold and Size Effects in Polyvinylpyrrolidone-Oxidized Single-Wall Carbon Nanohorn Nanocomposite: The Impact for Relative Humidity Resistive Sensors Design

**DOI:** 10.3390/s21041435

**Published:** 2021-02-19

**Authors:** Bogdan-Catalin Serban, Cornel Cobianu, Niculae Dumbravescu, Octavian Buiu, Marius Bumbac, Cristina Mihaela Nicolescu, Cosmin Cobianu, Mihai Brezeanu, Cristina Pachiu, Matei Serbanescu

**Affiliations:** 1 National Institute for Research and Development in Microtechnologies-IMT Bucharest, 126 A Erou Iancu Nicolae Str., 077190 Voluntari, Romania; cornel.cobianu@imt.ro (C.C.); niculae.dumbravescu@imt.ro (N.D.); cristina.pachiu@imt.ro (C.P.); 2 Research Center for Integrated System, Nanotechnologies, Carbon-Based Nanomaterials (CENASIC)-IMT, 126 A Erou Iancu Nicolae Str., 077190 Voluntari, Romania; 3 Academy of Romanian Scientists, Science, Technology of Information Section, 3 Ilfov Str., 077160 Bucharest, Romania; 4 Faculty of Sciences and Arts, Sciences and Advanced Technologies Department, Valahia University of Targoviste, 13 Sinaia Alley, 130004 Targoviste, Romania; marius.bumbac@valahia.ro; 5 Institute of Multidisciplinary Research for Science Technology, Valahia University of Targoviste, 13 Sinaia Alley, 130004 Targoviste, Romania; cristina.nicolescu@valahia.ro; 6 Electrical Engineering, Electronics and Information Technology Faculty, Valahia University of Targoviste, 13 Sinaia Alley, 130004 Targoviste, Romania; cosmincobianu@gmail.com; 7 Faculty of Electronics, University Politehnica of Bucharest Telecommunications and Information Technology, 1–3 Iuliu Maniu Blvd., 6th District, 061071 Bucharest, Romania; scriemiceva@hotmail.com (M.B.); matei.serbanescu@stud.etti.upb.ro (M.S.)

**Keywords:** oxidized carbon nanohorns (CNHox), polyvinylpyrrolidone (PVP), nanocomposite, relative-humidity resistive sensors, electrical percolation threshold, size effects, swelling, Young modulus elasticity

## Abstract

This paper reports, for the first time, on the electrical percolation threshold in oxidized carbon nanohorns (CNHox)–polyvinylpyrrolidone (PVP) films. We demonstrate—starting from the design and synthesis of the layers—how these films can be used as sensing layers for resistive relative humidity sensors. The morphology and the composition of the sensing layers are investigated through Scanning Electron Microscopy (SEM), Atomic Force Microscopy (AFM), and RAMAN spectroscopy. For establishing the electrical percolation thresholds of CNHox in PVP, these nanocomposite thin films were deposited on interdigitated transducer (IDT) dual-comb structures. The IDTs were processed both on a rigid Si/SiO_2_ substrate with a spacing of 10 µm between metal digits, and a flexible substrate (polyimide) with a spacing of 100 µm. The percolation thresholds of CNHox in the PVP matrix were equal to (0.05–0.1) wt% and 3.5 wt% when performed on 10 µm-IDT and 100 µm-IDT, respectively. The latter value agreed well with the percolation threshold value of about 4 wt% predicted by the aspect ratio of CNHox. In contrast, the former value was more than an order of magnitude lower than expected. We explained the percolation threshold value of (0.05–0.1) wt% by the increased probability of forming continuous conductive paths at much lower CNHox concentrations when the gap between electrodes is below a specific limit. The change in the nanocomposite’s longitudinal Young modulus, as a function of the concentration of oxidized carbon nanohorns in the polymer matrix, is also evaluated. Based on these results, we identified a new parameter (i.e., the inter-electrode spacing) affecting the electrical percolation threshold in micro-nano electronic devices. The electrical percolation threshold’s critical role in the resistive relative-humidity sensors’ design and functioning is clearly emphasized.

## 1. Introduction

The transport properties of composite materials, particularly those related to electrical transport, have been intensively investigated over the past 20 years. Conductive fillers (such as carbon fiber, carbon nanotubes, carbon black, etc.), incorporated in an insulating polymer can reduce the overall resistivity by several orders of magnitude by developing an infinite conductive path throughout the nanocomposite [1,2,3]. The transition from an insulating polymer to a conducting nanocomposite, as a function of the electrically conductive filler concentration, is defined as percolation. Furthermore, the lowest filler concentration value at which the insulating material is converted into a conductive composite is called percolation threshold, *ψc* [4,5].

Thanks to their high electrical conductivity and outstanding mechanical properties, the electrical percolation threshold of polymeric composites incorporating carbon nanotubes (CNTs) have received particular attention. This important characteristic of the nanocomposite depends on many parameters; among them, one can mention: the CNT type (SWCNT–single-walled carbon nanotube, MWCNT–multi-walled carbon nanotube), the synthesis method (either chemical vapor deposition, arc discharge, or laser ablation), the type of pre-chemical treatment (thermal oxidation, UV/O3, HNO3), functionalization (NH2, polyphenylene ethynylene), size and geometry of aggregates, polymer class, etc. [6,7,8,9,10]. Finally, the type of filler dispersion in the insulating matrix can influence the value of the percolation threshold. Fast stirring leads to statistical percolation (a process associated with a random distribution of the electrically conductive filler particles). In contrast, slow or medium stirring yields kinetic percolation (the particles are free to move and thereby can form a conducting network at much lower particle concentrations) [11].

In particular, the electrical threshold percolation is of great practical interest for applications directed to gas sensing [12].

Single-walled carbon nanohorns (SWCNHs), a kind of 0D carbon nano-allotrope consisting of flower-like architecture with sp^2^-hybridized carbon, were first reported by Iijima in 1999 [13]. These nanostructures exhibit remarkable properties: a simple synthesis procedure, excellent electrical conductivity (from 10 S/m to several hundred S/m), availability for covalent and noncovalent functionalization, fast mass transport, large specific surface area, and high porosity [14]. Based on these outstanding properties, the SWCNHs have been considered for different applications, such as drug-delivery system carriers, capacitors, composite materials, and gas adsorption [15,16,17,18,19]. However, little information about the gas-sensing capabilities of SWCNHs and their nanocomposites is available [20].

In the last years, sensing layers based on carbon nanohorns, oxidized carbon nanohorns, and their nanocomposites were also considered for the design and development of resistive sensors for ammonia [21], relative humidity [22,23,24,25,26,27], and ethanol [28,29,30,31,32] detection. 

Even though the addition of CNHs to dielectric or conductive polymers significantly increases the conductivity of the matrix nanocomposite [33], the information in scientific literature regarding the electrical percolation threshold of carbon nanohorns and their derivatives in different nanocomposites is scarce [34].

Bera et al. reported the preparation of nanocomposites based on carbon nanohorns (CNHs)/graphene nanoplates (GNP)/polystyrene (PS). These nanocomposites were synthesized through a two-step process: (a) in situ bulk polymerization of styrene monomer in the presence of CNHs, followed by (b) the addition of suspension polymerized GNP/PS bead during polymerization of styrene. The formation of continuous GNP–CNH–GNP, CNH–GNP–CNH, or CNH–CNH conductive paths, throughout the polystyrene matrix, was found at an exceptionally low loading of CNH (1.0 wt%) and GNP (0.15 wt%) [35].

A CNHs/epoxy nanocomposite was synthesized by Montes et al. The procedure involved mixing different amounts of the carbon conductive filler through the insulating polymeric matrix (from 2 to 10% w/w in CNHs). Percolation threshold was found at 5.5% CNH loading (w/w) [36].

Sedelnikova et al. explored the electromagnetic properties of polystyrene composites filled with CNHs. They observed percolation-like critical behaviors for low-frequency permittivity and conductivity, as well as for the microwave electromagnetic response of composites [37].

Fraczek-Szczypta et al. showed that 1 wt% amount of SWCNHs could form the electrical conductivity mesh in a polyacrylonitrile matrix [38].

The addition of a conductive filler in an insulating matrix affects their mechanical properties, also [39]. For example, Reffaee et al. studied the electrical and mechanical properties of nanocomposites based on linear low-density polyethylene/high abrasion furnace black and acrylonitrile rubber/high abrasion furnace black with a different fraction of electrically conductive filler to find out the percolation thresholds. The mechanical properties for both nanocomposites indicated a sharp increase at the same concentration of conductive filler found in the case of electrical measurements [40].

This paper investigates the electrical statistical percolation threshold, *ψc,* for an oxidized carbon nanohorns (CNHox)-polyvinylpyrrolidone (PVP) nanocomposite. The statistical percolation threshold is a critical parameter needed to investigate and further optimize this nanocarbon matrix’s resistive sensing capabilities towards water molecules. Moreover, the mechanical properties of nanocomposites at the percolation threshold are discussed.

## 2. Materials and Methods

**Figure 1:**
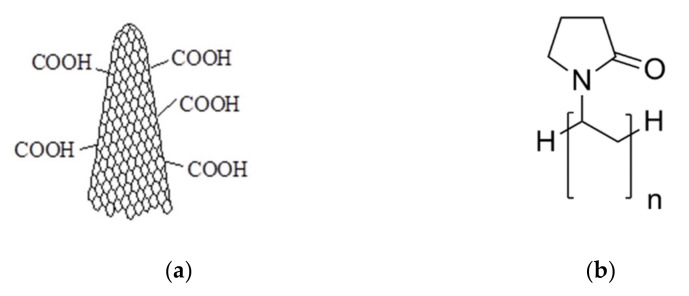
The structure of (**a**) oxidized carbon nanohorn (**CNHox**) and (**b**) polyvinylpyrrolidone (PVP).

The oxidized carbon nanohorns (**CNHox** powder, 0% metallic compounds, 10% graphite), with a diameter 2–5 nm (TEM) and a length of 40–50 nm, having a specific surface area around 1300–1400 m^2^/g (BET), were purchased from Sigma Aldrich (Sigma-Aldrich, Taufkirchen City, Germany) [41] and are depicted in Figure 1a. Polyvinylpyrrolidone (PVP) with an average molecular weight of 360,000 (see Figure 1b) and isopropyl alcohol (IPA) were purchased from Sigma Aldrich, also. Both reagents were of analytical grade and used “as received” without further purification.

**Figure 2:**
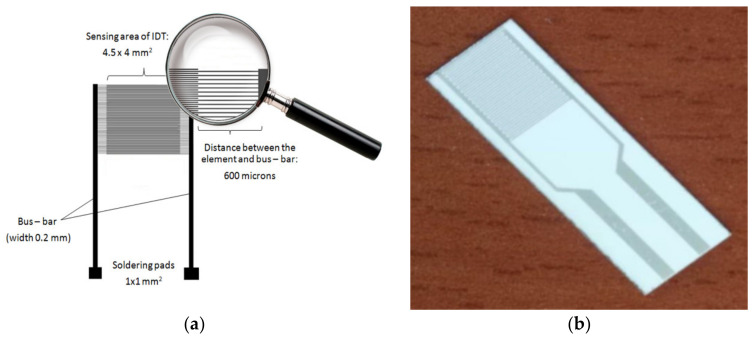
The geometry of: (**a**) interdigitated transducer (IDT) on rigid substrate (Si/SiO_2_) and (**b**) IDT on flexible substrate (polyimide).

For the investigation of the electrical percolation threshold, we used Inter DigitatedTransducer (IDT) structures fabricated both on a rigid (Figure 2a) and flexible substrate (Figure 2b).

The rigid IDT dual-comb structures were manufactured on a Si substrate (470 μm thickness), covered by a SiO_2_ layer (1 μm thickness). The metal stripes of IDT comprised Cr (10 nm thickness) and Au (100 nm thickness). The width of the metal digit was 10 μm, with a 0.6 mm separation between the collecting electrodes (bus-bar with a width of 0.2 mm) (Figure 2a). The authors purchased the flexible IDT dual-comb structures (gold interdigitated electrodes made of transparent flexible polyimide plastic substrate with digit width and spacing equal to 100 µm) from Dropsens (Metrohm DropSense, Oviedo, Spain) [42].

The nanocomposite films were synthesized according to the following procedure [43]:

**CNHox**/PVP dispersions were prepared in water–isopropyl alcohol mixtures (1/1, v/v). The homogenization of the dispersions was achieved by employing a mild sonication bath (FS20D Fisher Scientific, Dreieich, Germany), at 42 kHz (output power 70 W), for 30 min. This process ensured a uniform dispersion of the **CNHox** in the PVP matrix, suitable for a statistical percolation approach.

The obtained dispersion was deposited on both the rigid substrate (Figure 2a) and the flexible substrate (Figure 2b) by the drop-casting method. The dispersions were prepared so that the concentration of **CNHox** in the matrix nanocomposite deposited on the substrate varied between 0.1 wt% and 10 wt%.

The Raman spectra have been collected at room temperature with a Witec Raman spectrometer (Alpha-SNOM 300 S, WiTec. GmbH, Germany) using the 532 nm line, with a maximum power of 145 mW. The incident laser beam with a spot-size of about 1.0 µm was focused onto the sample with a 100× long-working distance microscope objective. The Raman spectra were measured with an exposure time of 20 s accumulation and the scattered light was collected by the same objective in back-scattering geometry with 600 grooves/mm grating. The Raman systems’ calibration was carried out using the 512 cm^−1^ Raman line of a silicon substrate (Figure 6-black line spectra), which corresponds to the longitudinal optical-transverse optical (LO-TO) phonon. The spectrometer scanning data collection and processing were carried out by a dedicated computer using WiTec Project Five software. 

The Scanning Electron Microscopy (SEM) was performed on TESCAN VEGA II LMU-General Purpose equipment (resolution: 3 nm at 30 kV, accelerating voltage 200 V–30 kV, electron gun source: tungsten filament, magnification: 13×–1,000,000×, detectors: SE, BSE, LVSTD) (TESCAN ORSAY HOLDING, a.s., Brno, Czech Republic).

The AFM measurements were performed using a NanoSurf EasyScan series 2 instrument (Nanosurf AG, Liestal, Switzerland).

## 3. Results

### 3.1. Morphological and Compositional Characterization of the CNHox and PVP Nanocomposite

**Figure 3:**
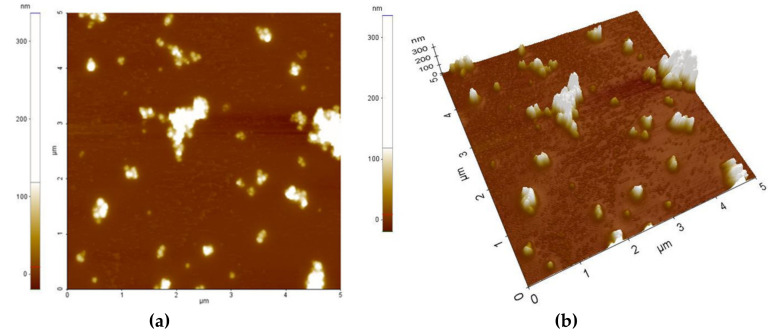
Atomic Force Microscopy (AFM) images of 1 wt% oxidized single-walled carbon nanohorns (ox-SWCNHs) in the PVP matrix: (**a**) top view and (**b**) perspective view.

**Figure 4:**
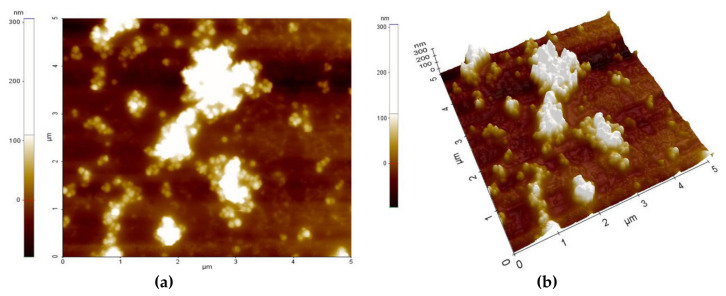
AFM images of 2 wt% ox-SWCNHs in the PVP matrix: (**a**) top view and (**b**) perspective view.

Figure 3 and Figure 4 present the AFM topography of the thin films of the PVP/CNHox nanocomposite, as follows: with 1 wt% CNHox mass concentration (Figure 3) and 2 wt% CNHox (Figure 4). For both cases, the scanned area was 5 × 5 μm^2^.

**Figure 5:**
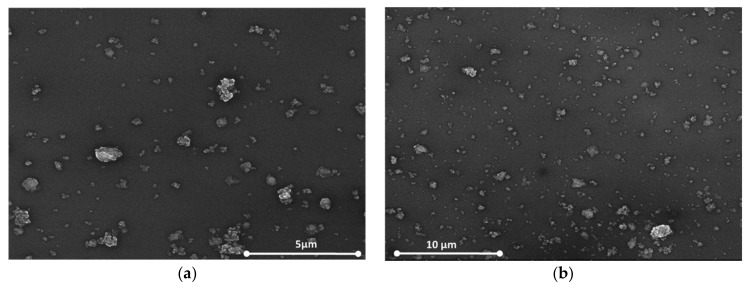
The film’s Scanning Electron Microscopy (SEM) image containing: (**a**) 1 wt% **CNHox** in PVP and (**b**) 2 wt% CNHox in PVP.

Figure 5a,b show the SEM images for the same nanocomposites with 1 wt% concentration of oxidized single-walled carbon nanohorns (ox-SWCNHs) in PVP (Figure 5a) and 2 wt% ox-SW**CNHs** in PVP (Figure 5b). As expected, for these relatively small differences in the amounts of **CNHox** incorporated in the PVP matrix, minor texture differences can be identified in the AFM and SEM images, as a function of carbonic loading.

**Figure 6:**
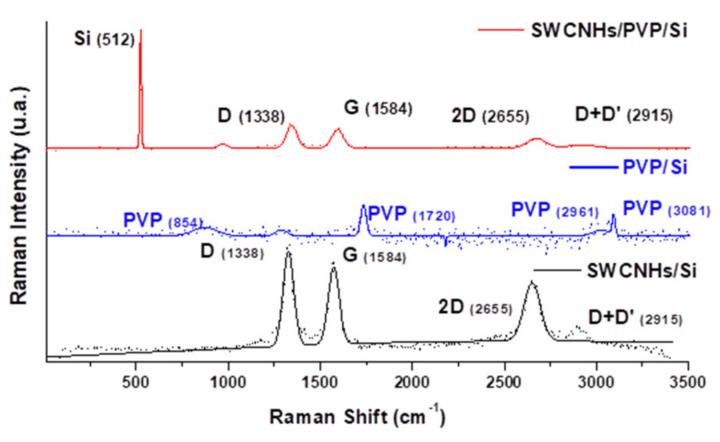
The Raman spectra for (top to bottom): (**top**) 2 wt% of ox-SWCNH in the PVP matrix; (**middle**) PVP on Si; (**bottom**) ox-SWCNH.

The measured Raman spectrum of the **CNHox**–PVP nanocomposite (2 wt%) is depicted in Figure 6 top. Figure 6 bottom presents the Raman of CNHox measured on test samples, while Figure 6 middle illustrates the Raman spectrum for PVP.

The recorded spectrum exhibited the typical D (~1329 cm^−1^), G (~1577 cm^−1^), and 2D (~2651 cm^−1^) modes, which are characteristic of carbon nanomaterials. The G mode corresponds to the graphite mesh’s tangential atomic vibrations, while the D mode reveals defect carbon states different from the graphite mesh. The spectrum for the synthesized CNHox/PVP showed Raman shifts due to the formation of hydrogen bonds between ox-SWCNH (D (~1315 cm^−1^), G (~1590 cm^−1^), and 2D (~2616 cm^−1^), D+G (2984 cm^−1^), and PVP modes [22]. Therefore, these Raman band shifts indicated the chemical interaction of the two materials inside the nanocomposite, thus formed, proving that we have indeed more than a mechanical mixture.

### 3.2. Determination of Electrical Percolation Threshold ψ_c_ for CNHox–PVP Films Deposited on a Rigid Substrate

**CNHox**–PVP films with concentration levels of the **CNHox** in the PVP matrix ranging from 0.01 wt% to 10 wt%, were deposited by drop-casting on the SiO_2_/Si rigid substrate IDT dual-comb structures with metal digits width and interdigit spacing of 10 µm. The films’ drying process was performed at room temperature for 24 h, and then the electrical resistance was measured. The experimental data revealed a significant decrease in the resistance that occurred at a concentration of 0.05 wt% of CNHox in the PVP matrix, which indicated that the nanocomposite’s percolation threshold (*ψ_c_*) was reached.

**Figure 7:**
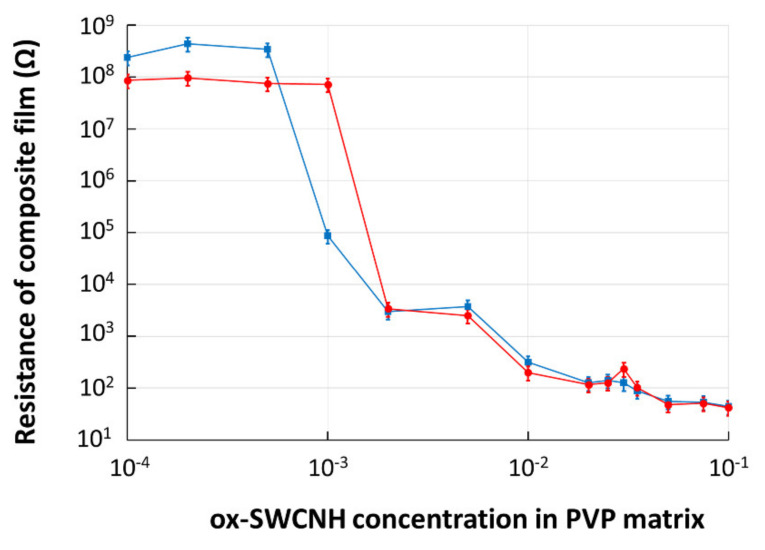
The electrical resistance of composite film deposited on rigid Si/SiO_2_ substrate versus mass concentration of ox-SWCNH in PVP matrix: in blue—data for “as dried” film; in red—data after annealing.

To investigate the annealing effects on the percolation threshold, the same samples were exposed to a heat treatment for 60 h at 110 °C and a pressure of 1 mbar. The samples’ electrical resistance was measured again, and the results are included in Figure 7. No change in the percolation threshold was recorded.

### 3.3. Determination of Electrical Percolation Threshold ψ_c_ for Oxidized Carbon Nanohorns–PVP Films Deposited on a Flexible Substrate

**Figure 8:**
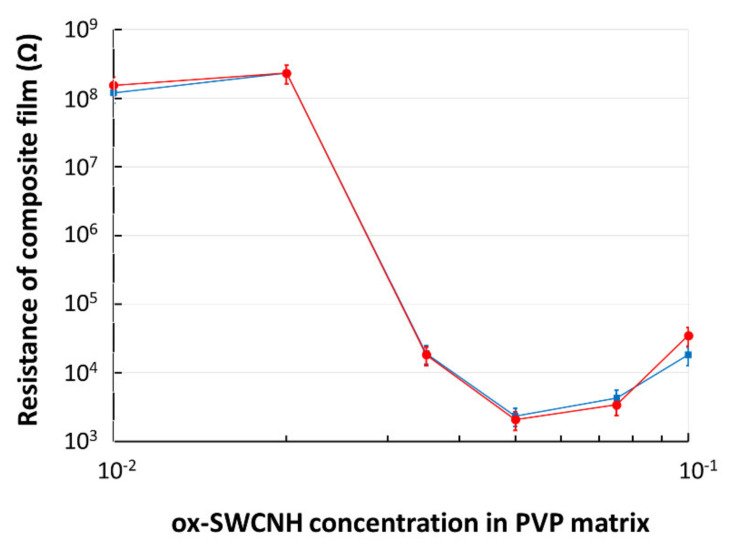
The electrical resistance of composite film deposited on a flexible substrate versus mass concentration (%) of ox-SWCNH in PVP. In blue--data for “as dried” film; in red—data after annealing.

The **CNHox**–PVP nanocomposite films were deposited on a flexible, plastic substrate containing IDT with metal digits width and spacing of 100 µm. The electrical resistance measurements for the “as dried” films (Figure 8) indicated a percolation threshold corresponding to a concentration of 3.5 wt% of the oxidized carbon nanohorns in the PVP matrix. After the heat treatment of the samples (110 °C, 1 mbar, 60 h), it was observed that the percolation threshold remained unchanged. These percolation results agree with the value of the electrical percolation threshold resulting from the geometry of oxidized carbon nanohorns, as explained below.

## 4. Discussion

According to the literature data [1], for a statistical distribution of oxidized carbon nanohorns, *ψc* ≈ 1/η, where *ψc* is electrical percolation threshold and η is the aspect ratio (the length-to-diameter ratio of CNHox), at a length of 50 nm and a diameter of 2 nm (“aspect ratio” = 25), the degree of percolation should be around 4 wt%, a value which is close to ***ψc = 3.5 wt%,*** which we have experimentally obtained for an IDT test structure with digit width and spacing of 100 µm. 

On the other hand, the percolation threshold (***ψc = (0.05–0.1) wt%***) for the **CNHox** in PVP film deposited on IDT of a rigid substrate was more than one order of magnitude smaller than the 1/η value. This result could be explained by the higher probability of **CNHox** forming percolating paths between two electrodes when the distance between them is so drastically reduced (digit width and spacing was, in this case, 10 µm, one order of magnitude lower than in the case of the flexible substrate). To the best of our knowledge, it is for the first time when the spacing between electrodes is identified as a critical parameter determining the percolation threshold value. This parameter plays an essential role in the design and fabrication of the micro-nano-electronic devices (in particular, for the resistive sensors), for which the operation is closely related to the percolation threshold of the carbonic materials within a polymeric matrix.

We also compared the experimental results we have obtained with a recent series of percolation threshold studies utilizing different nanocomposites polymers—carbon nano-allotrope-based conducting fillers, as shown in Table 1. As can be seen, the dispersion of the reported percolation threshold values is large (7 wt% to 0.006 wt%); at the same time, the information available about the specific design of the test structures employed is not sufficiently detailed.

The experimental study presented above has relevance on its own. Still, it was also motivated by the need to answer the following question: why is the identification of the electrical percolation threshold such a critical parameter in the evaluation of the resistive sensing capabilities of ox-SWCNH-PVP composite toward water molecules? To answer this question, one needs to explain the choice of this particular nanocomposite as a potential resistive sensitive layer for relative humidity (RH) detection. Both components of the nanocomposite appear to have appropriate properties for this purpose.

**Figure 9:**
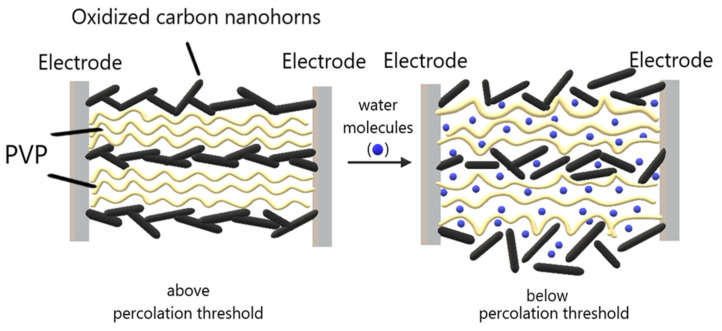
Swelling of hydrophilic CNHox–PVP nanohybrid around the electrical percolation threshold.

Firstly, oxidized carbon nanohorns are nanocarbonic materials with hydrophilic properties, uniform size, high conductivity, and large surface area. Secondly, PVP is an electrically insulating polymer, but that may become conductive, through percolation, by adding oxidized carbon nanohorns. Moreover, PVP is highly hygroscopic, film forming, and swells when absorbing moisture in humid environments. If the concentration of oxidized carbon nanohorns is only slightly higher than the percolation threshold, a small amount of swelling may cause a considerable change in sensor resistance (Figure 9) due to the increase of the width of the energy barrier for the tunneling of the charge carriers from one nanohorn to the near neighbor. Therefore, the sensors’ high sensitivity is achieved in the concentration range of oxidized carbon nanohorns corresponding to the percolation threshold.

If the concentration of conductive filler substantially exceeds the percolation threshold, the change in resistance (for different RH levels) can be described more or less by a linear expression. While these resistive sensors’ fabrication reproducibility is much better, the sensitivity is significantly lower [53,54,55].

Given the high relevance of carbon allotropes-based nanocomposites for various applications, such as sensing [34,36], energy storage [56], development of protective coatings [57], and membranes for wastewater treatment [58], there is an increased interest in the community in correlating the specific functional properties with the electrical and mechanical properties of the nanocomposite, while considering the type and specific geometrical properties of the constituents [59,60,61].

Our research is overall focused on the realization of RH sensors on both solid and flexible substrates; within this scope of work, the sensing layer might be realized through successive deposition steps (employing the “drop-casting” method), aiming at optimizing both the electrical transport properties and the thermomechanical ones. Following on our previously reported results [62], we concentrate in this paper on the correlation between the electrical percolation level and the change in the mechanical properties of the oxCNs–PVP nanocomposite films.

Several micromechanics models can be used to estimate the effective thermoelastic properties of the nanocomposite material. Among them, one could mention the Halphin–Tsai [63,64] and the modified Halphin–Tsai [65] models. The original Halphin–Tsai model (valid for isotropic constituents [66]) assumes that Young’s longitudinal module of the composite ($E_{c}^{l}$) is the weighted sum of two distinct contributions, coming from the “matrix material” ($E_{m}$) and the “filler material” ($E_{f}$):

(1) $$E_{c}^{l}=E_{f}v_{f}+E_{m}v_{m}$$

where $v_{f}$ and $v_{m}$ are the volume fractions for the “filler material” and “matrix material,” respectively. The transversal component of Young’s module of the composite ($E_{c}^{t}$) can be written as:

(2) $$E_{c}^{t}=E_{m}\frac{1+\xi \eta v_{f}}{1-\eta v_{f}}$$

where *ξ* is called the reinforcing factor. Reference [66] also indicates how this reinforcing factor can be calculated for various geometries and a given type of elastic module (transversal or longitudinal). In particular, for a circular or rectangular fiber, characterized by a length L in one direction and thickness or a diameter T or D, the reinforcing factor is provided by the formula:

(3) $$E_{c}^{t}=E_{m}\frac{1+\xi \eta v_{f}}{1-\eta v_{f}}$$

While *η* can be calculated as follows:

(4) $$\eta =\frac{\left(E_{f}/E_{m}\right)-1}{\left(E_{f}/E_{m}\right)+\xi }$$

The term provided by Equation (3) is obviously an “aspect ratio” factor, which is also taken into account in reference [60], which suggests an inverse connection between the percolation threshold (φ_p_) and the aspect ratio of CNTs (α = L/d), where L is their length and d is the diameter of CNTs, respectively. By neglecting the geometrical factors related to the matrix, the elastic moduli of the carbon nanocomposite can be written as [66]:

(5) $$E=\frac{3}{8}E_{L}+\;\frac{5}{8}E_{T}$$

where the two components (longitudinal—*E_L_* and transversal—*E_T_*) can be calculated as follows:

(6) $$E_{L}=E_{m}\frac{1+2\alpha \eta_{L}\varphi_{f}}{1-\eta_{L}\varphi_{f}}$$

(7) $$E_{T}=E_{m}\frac{1+2\alpha \eta_{T}\varphi_{f}}{1-\eta_{T}\varphi_{f}}$$

And $\varphi_{f}$ is the volume fraction of modifier (i.e., the carbon allotrope) within the matrix. The longitudinal and transversal factors ($\eta_{L}\;\mathrm{and}\;\eta_{T}$) can be calculated based on the following equations:

(8) ηL=EfEm−1EfEm+2α

(9) ηT=EfEm−1EfEm+2

where $E_{f}$ is the elastic moduli of the nanoparticles, while $E_{m}$ is the elastic moduli of the polymer matrix. One crucial observation; it can be seen that only the longitudinal coefficient $\eta_{L}$ depends on the percolation threshold ($\varphi_{P}$). Based on the above formulas, one can calculate the longitudinal component of the elastic module (*E_L_*) for the nanocomposite, based on the elastic properties of the constituents, the experimentally determined percolation threshold, and the volume fraction of the oxidized carbon nanohorns: 

To quantify the change in the elastic module of the polymer matrix due to the presence of a certain quantity of oxidized carbon nanohorns, one has to address a couple of issues, as follows:

A. The elastic module of the carbon nanohorns.

A vast majority of the papers published suggest that the mechanical properties of the carbon nanohorns should be considered as very similar to those of the carbon nanotubes (CNTs). Kumar et al. [67] developed a computational methodology and calculated the elastic module as a function of the aspect ratio and the apex angle of the carbon nanohorn (where carbon nanotubes can be seen as a particular case of a carbon nanohorns with zero conical apex angle). The obtained values varied between 375 GPa (for a 19.2° angle) and 250 GPa (for a 60° angle) for a carbon nanohorn with a diameter of 2.4 nm and an aspect ratio (length/radius) equal to 6. How does this compare with the published data on CNTs? Treacy et al. [68] report an average value of 1800 GPA for the elastic modulus; in a more recent contribution, Deng et al. reported an effective elastic module ranging between 530 and 700 GPa for single-walled carbon nanotubes [69]. The authors claimed that these values—much lower than those previously reported—were due to bundling and finite-length effects. A study employing molecular dynamics for studying the temperature effects upon the elastic properties of SWCNTs reported a value of 401 GPa for the Young module [70]. In Yakobson’s and Avouris’s work, a review of the experimental results related to carbon nanotubes’ mechanical properties was conducted, also indicating how the Young module should scale with regards with the single-wall carbon nanotube diameter, d: E = (1.36 − 1.76) TPa nm/d [71]. If one considers the diameter range for the oxCNHs, indicated by the supplier (i.e., 2–5 nm), this results in an elastic module ranging from 272 GPa to 880 GPa.

B. The elastic module of the polymer (PVP) matrix.

PVP-based films were investigated as potential binders for tableting films; the mechanical properties of such films were investigated, and significant dependence of the elastic module on the humidity level was reported: from 0.93 to 0.46 GPa, for a relative humidity variation from 20% to 65% [72]. PVP fibers were also synthesized to develop fiber-oriented membranes [73], and an elastic module varying between 165 MPa and 71 MPa was reported.

C. Finally, we will assume that the conversion between the concentration (at threshold), which is expressed in weight %, and the volume % associated with a particular filler concentration, is used in [1], i.e., vol% = wt% for single-wall nanotubes.

**Figure 10:**
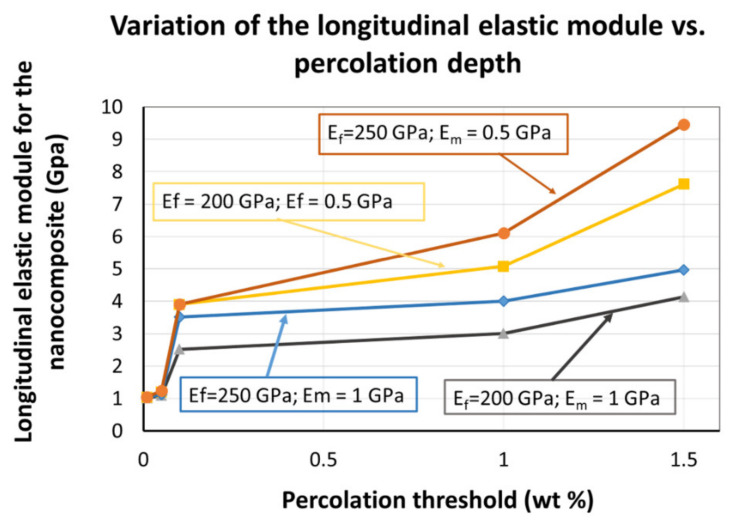
The nanocomposite’s longitudinal elastic module (EL) variation, as a function of electrical percolation threshold (experimentally determined in this work).

In Figure 10, we are presenting the results of the calculations, i.e., the longitudinal elastic module of the nanocomposite, for five different percolation levels and considering for each of the two constituents two sets of values: 200 and 250 GPa for the oxidized carbon nanohorns and 1 and 0.5 GPa for PVP. The calculations do include the percolation level recorded within our experiments (0.05–0.1) wt%, recorded for the nanocomposite deposited on the rigid substrate. In this region, an abrupt change in the value of the longitudinal elastic module is registered.

## 5. Conclusions

This paper reports on the design and synthesis of oxidized single-wall carbon nanohorns–polyvinylpyrrolidone films as potential sensing layers for resistive relative humidity sensing. The sensing layers’ morphology and qualitative composition were investigated through Atomic Force Microscopy (AFM), Raman spectroscopy, and Scanning Electron Microscopy (SEM). 

We experimentally determined the percolation threshold of oxidized carbon nanohorns in polyvinylpyrrolidone (PVP) by depositing the nanocomposite films on interdigitated transducers (IDT) dual-comb structures both on a rigid Si/SiO_2_ substrate and on a flexible substrate (polyimide). The percolation threshold was achieved at a concentration of (0.05–0.1) wt% of CNHox in the nanocomposite matrix for the layers deposited on the rigid substrate. In the case of flexible IDTs, the percolation threshold had a value of 3.5 wt%. The latter value agrees with the prediction given by the aspect ratio of the CNHox. The difference of about an order of magnitude between the experimental results can be due to the fact that the rigid substrate had a spacing between the IDT electrodes of 10 µm, compared with to the flexible substrate, which had a spacing of 100 µm between the IDT electrodes. Thus, a new parameter for the value of percolation threshold of nanocarbonic materials, distance between IDTs electrodes, was identified. This conclusion may influence the design and fabrication of micro-nano-electronic devices operating in correlation with the percolation threshold.

The importance of identifying the electrical percolation threshold as a critical parameter in the evaluation of resistive sensing capabilities of CNHox–PVP towards water molecules is demonstrated, also. The results related to the electrical percolation threshold were also used to evaluate the enhancement of the nanocomposite’s mechanical properties, as reflected by the changes in the elastic module. Using a formalism well accepted in the literature, a change in the elastic module between 10–17% was recorded for the electrical percolation threshold r. These results have a potential impact in supporting further work to optimize the sensing structures (better response time, reduced hysteresis, mechanical stability) to be developed on flexible substrates.

## Figures and Tables

**Table 1:** Comparison of percolation threshold in different nanocomposites based on carbonic filler embedded in a polymeric matrix, as a function of the synthesis method.

Conductive Carbonic Filler	Inert Polymeric Matrix	Synthesis Method	Percolation Threshold (wt%)	Reference
SWCNT	Epoxy	Dispersion (sonicated, heat sheared)	0.05	[44]
Large-area reduced graphene oxide (LrGO)	Polyvinyl alcohol (PVA)	Dispersion (stirred at 98 °C for 30 min)	0.189	[45]
Carbon nanotubes	High-density polyethylene	Melt processing technique	4	[46]
MWCNT	Polystyrene latex	Dispersion in water (sonication for 30 min at room temperature)	4	[47]
MWCNT	PVC	Dispersion (stirred, grinded, hot-pressed)	0.094	[48]
MWCNT	P_3_HT/PMMA	Dispersion in trifluoroacetic acid/tetrahydrofurane (sonicated)	0.006	[49]
MWCNT	PET	Coagulation method	0.9	[50]
MWCNT	PS-latex	Dispersion in water, sonicated	0.36	[51]
Thermally reduced graphene oxide	High-density polyethylene- polypropylene	Melt compounding method	3	[52]
Surfactant exfoliated graphene	High-density polyethylene- polypropylene	Melt compounding method	7	[52]

## Data Availability

The data presented in this study are available within the present article.

## References

[1] Bauhofer W., Kovacs J.Z. (2009). A review and analysis of electrical percolation in carbon nanotube polymer composites. Compos. Sci. Technol..

[2] Khan W.S., Asmatulu R., Eltabey M.M. (2013). Electrical and thermal characterization of electrospun PVP nanocomposite fibers. J. Nanomater..

[3] Kovacs J.Z., Velagala B.S., Schulte K., Bauhofer W. (2007). Two percolation thresholds in carbon nanotube epoxy composites. Compos. Sci. Technol..

[4] Sandler J., Kirk J.E., Kinloch I.A., Shaffer M., Windle A.H. (2003). Ultra-low electrical percolation threshold in carbon-nanotube-epoxy composites. Polymer.

[5] Lu C., Mai Y.W. (2008). Anomalous electrical conductivity and percolation in carbon nanotube composites. J. Mater. Sci..

[6] Kim B.J., Lee I.Y. (2003). Electrical properties of single-wall carbon nanotube and epoxy composites. J. App. Phys..

[7] Li J., Ma P.C., Chow W.S., To C.K., Tang B.Z., Kim J.-K. (2007). Correlations between percolation threshold, dispersion state, and aspect ratio of carbon nanotubes. Adv. Funct. Mater..

[8] Antonucci V., Faiella G., Giordano M., Nicolais L., Pepe G. (2007). Electrical properties of single walled carbon nanotube reinforced polystyrene composites. Macromol. Symp..

[9] Ramasubramaniam R., Chen J., Liu H. (2003). Homogeneous carbon nanotube/polymer composites for electrical applications. Appl. Phys. Lett..

[10] Nogales A., Broza G., Roslaniec Z., Schulte K., Šics I., Hsiao B.S., Ezquerra T.A. (2004). Low percolation threshold in nanocomposites based on oxidized single wall carbon nanotubes and poly (butylene terephthalate). Macromolecules.

[11] Dang Z.-M., Shehzad K., Zha J.-W., Mujahid A., Hussain T., Nie J., Shi C.-Y. (2011). Complementary percolation characteristics of carbon fillers based electrically percolative thermoplastic elastomer composites. Compos. Sci. Technol..

[12] Pandis C., Peoglos V., Kyritsis A., Pissis P. (2011). Gas sensing properties of conductive polymer nanocomposites. Procedia Eng..

[13] Iijima S., Yudasaka M., Yamada R., Bandow S., Suenaga K., Kokai F., Takahashi K. (1999). Nano-aggregates of single-walled graphitic carbon nano-horns. Chem. Phys. Lett..

[14] Zhu S., Xu G. (2010). Single-walled carbon nanohorns and their applications. Nanoscale.

[15] Serban B.C., Bumbac M., Buiu O., Cobianu C., Brezeanu M., Nicolescu C. (2018). Carbon nanohorns and their nanocomposites: Synthesis, properties, and applications. A concise review. Ann. Acad. Rom. Sci. Ser. Math. Appl..

[16] Sani E., Mercatelli L., Barison S., Pagura C., Agresti F., Colla L.M., Sansoni P. (2011). Potential of carbon nanohorn-based suspensions for solar thermal collectors. Sol. Energy Mater. Sol. Cells.

[17] Ajima K., Yudasaka M., Murakami T., Maigné A., Shiba K., Iijima S. (2005). Carbon nanohorns as anticancer drug carriers. Mol. Pharm..

[18] Karousis N., Suarez-Martinez I., Ewels C.P., Tagmatarchis N. (2016). Structure, properties, functionalization, and applications of carbon nanohorns. Chem. Rev..

[19] Marinescu R., Serban B.-C., Dumbravescu N., Avramescu V., Cobianu C., Buiu O. (2019). Carbon-Based materials for healthcare microdevices. Nonconv. Tech. Rev./Revista de Tehnologii Neconventionale.

[20] Suehiro J., Sano N., Zhou G., Imakiire H., Imasaka K., Hara M. (2006). Application of dielectrophoresis to fabrication of carbon nanohorn gas sensor. J. Electrost..

[21] Sano N., Kinugasa M., Otsuki F., Suehiro J. (2007). Gas sensor using single-wall carbon nanohorns. Adv. Powder Technol..

[22] Serban B.C., Buiu O., Dumbravescu N., Cobianu C., Avramescu V., Brezeanu M., Bumbac M., Nicolescu C.M. (2020). Oxidized carbon nanohorns as novel senasing layer for resistive humidity sensor. Acta Chim. Slov..

[23] Șerban B.C., Buiu O., Cobianu C., Avramescu V., Dumbrăvescu N., Brezeanu M., Bumbac M., Nicolescu C.M., Marinescu R. (2019). Ternary carbon-based nanocomposite as sensing layer for resistive humidity sensor. Proceedings.

[24] Șerban B.C., Buiu O., Cobianu C., Avramescu V., Dumbrăvescu N., Pachiu I.C., Ionescu O.N., Marinescu M.R. (2020). Chemiresistive Humidity Sensor Based on Matrix Nanocomposite Containing Hydrophilic Carbon Nanohorns. Patent RO.

[25] Șerban B.C., Cobianu C., Buiu O., Dumbrăvescu N., Avramescu V., Brezeanu M., Marinescu M.R. Ternary oxidized carbon nanohorn-based nanohybrid as sensing layer for resistive humidity sensor. Proceedings of the 3rd International Conference on Emerging Technologies in Materials Engineering.

[26] Șerban B.C., Cobianu C., Buiu O., Dumbrăvescu N., Avramescu V., Brezeanu M., Marinescu M.R. Ternary hydrophilic carbon nanohorn/ZnO/PVP nanohybrid structure for room temperature resistive humidity sensing. Proceedings of the 3rd International Conference on Emerging Technologies in Materials Engineering.

[27] Selvam K.P., Nakagawa T., Marui T., Inoue H., Nishikawa T., Hayashi Y. (2020). Synthesis of solvent-free conductive and flexible cellulose–carbon nanohorn sheets and their application as a water vapor sensor. Mater. Res. Express.

[28] Șerban B.-C., Buiu O., Cobianu C., Ionescu O., Varsescu D., Marinescu R., Dumbravescu N. (2019). Ethanol Sensor and Process for Preparing the Same. Romanian Patent Application.

[29] Șerban B.-C., Buiu O., Cobianu C., Ionescu O., Varsescu D., Marinescu R., Dumbravescu N. (2019). Chemiresistive Ethanol Sensor. Romanian Patent Application.

[30] Șerban B.-C., Buiu O., Cobianu C., Ionescu O., Varsescu D., Avramescu V., Marinescu R., Dumbravescu N. (2019). Sensitive Layer for Ethanol Sensor and Process for Manufacturing the Same. Romanian Patent Application.

[31] Cobianu C., Șerban B.-C., Dumbravescu N., Buiu O., Avramescu V., Pachiu C., Bita B., Bumbac M., Nicolescu C.-M., Cobianu C. (2020). Organic–inorganic ternary nanohybrids of single-walled carbon nanohorns for room temperature chemiresistive ethanol detection. Nanomaterials.

[32] Cobianu C., Serban B.-C., Dumbravescu N., Buiu O., Avramescu V., Bumbac M., Nicolescu C.-M., Cobianu C. Room temperature chemiresistive ethanol detection by ternary nanocomposites of oxidized single wall carbon nanohorn (ox-SWCNH). Proceedings of the 2020 International Semiconductor Conference (CAS).

[33] Tao Y., Noguchi D., Yang C.-M., Kanoh H., Tanaka H., Yudasaka M., Iijima S., Kaneko K. (2007). Conductive and mesoporous single-wall carbon nanohorn/Organic aerogel composites. Langmuir.

[34] Costa P., Nunes-Pereira J., Oliveira J., Silva J., Moreira J.A., Carabineiro S., Buijnsters J., Lanceros-Mendez S. (2017). High-performance graphene-based carbon nanofiller/polymer composites for piezoresistive sensor applications. Compos. Sci. Technol..

[35] Bera R., Suin S., Maiti S., Shrivastava N.K., Khatua B.B. (2015). Carbon nanohorn and graphene nanoplate based polystyrene nanocomposites for superior electromagnetic interference shielding applications. J. Appl. Polym. Sci..

[36] Montes R., Baeza M., Muñoz J. (2020). 0D polymer nanocomposite carbon-paste electrodes using carbon nanohorns: Percolating networks, electrochemical achievements and filler comparison. Compos. Sci. Technol..

[37] Sedelnikova O.V., Baskakova K.I., Gusel’Nikov A.V., Plyusnin P.E., Bulusheva L.G., Okotrub A.V. (2019). Percolative composites with carbon nanohorns: Low-frequency and ultra-high frequency response. Materials.

[38] Fraczek-Szczypta A., Blazewicz S. (2011). Manufacturing and physico-mechanical characterization of carbon nanohorns/polyacrylonitrile nanocomposites. J. Mater. Sci..

[39] Nikfar N., Zare Y., Rhee K.Y. (2018). Dependence of mechanical performances of polymer/carbon nanotubes nanocomposites on percolation threshold. Phys. B Condens. Matter.

[40] Reffaee A.S., El Nashar D., Abd-El-Messieh S., Nour K.A.-E. (2009). Electrical and mechanical properties of acrylonitrile rubber and linear low density polyethylene composites in the vicinity of the percolation threshold. Mater. Des..

[41] Carbon Nanohorns, Oxidized. https://www.sigmaaldrich.com/catalog/product/aldrich/804126?lang=en&region=RO.

[42] Interdigitated Electrodes/Microelectrodes. http://www.dropsens.com/en/interdigitated_electrodes.html.

[43] Serban B.-C., Cobianu C., Dumbravescu N., Buiu O., Avramescu V., Bumbac M., Nicolescu C.-M., Cobianu C., Brezeanu M. Electrical percolation threshold in oxidized single wall carbon nanohorn-polyvinylpyrrolidone nanocomposite: A possible application for high sensitivity resistive humidity sensor. Proceedings of the 2020 International Semiconductor Conference (CAS).

[44] Moisala A., Li Q., Kinloch I.A., Windle A.H. (2006). Thermal and electrical conductivity of single-and multi-walled carbon nanotube-epoxy composites. Compos. Sci. Technol..

[45] Zhou T.N., Qi X.D., Fu Q. (2013). The preparation of the poly(vinyl alcohol)/graphene nanocomposites with low percolation threshold and high electrical conductivity by using the large-area reduced graphene oxide sheets. Express Polym. Lett..

[46] Zhang Q., Rastogi S.S., Chen D., Lippits D., Lemstra P.J. (2006). Low percolation threshold in single-walled carbon nanotube/high density polyethylene composites prepared by melt processing technique. Carbon.

[47] Uğur Ş., Yargi Ö., Pekcan Ö. (2010). Conductivity percolation of carbon nanotubes (CNT) in polystyrene (PS) latex film. Can. J. Chem..

[48] Mamunya Y., Boudenne A., Lebovka N., Ibos L., Candau Y., Lisunova Y.M. (2008). Electrical and thermophysical behavior of PVC-MWCNT nanocomposites. Compos. Sci. Technol..

[49] Chen H., Muthuraman H., Stokes P., Zou J., Liu X., Wang J., Huo Q.I., Khondaker S., Zhai L. (2007). Dispersion of carbon nanotubes and polymer nanocomposite fabrication using trifluoroacetic acid as a co-solvent. Nanotechnology.

[50] Hu G., Zhao C., Zhang S., Yang M., Wang Z. (2006). Low percolation thresholds of electrical conductivity and rheology in poly(ethylene terephthalate) through the networks of multi-walled carbon nanotubes. Polymer.

[51] Dalmas F., Cavaillé J.Y., Gauthier C., Chazeau L., Dendievel R. (2007). Viscoelastic behavior and electrical properties of flexible nanofiber filled polymer nanocomposites. Influence of processing conditions. Compos. Sci. Technol..

[52] Tu C., Nagata K., Yan S. (2015). Morphology and electrical conductivity of polyethylene/polypropylene blend filled with thermally reduced graphene oxide and surfactant exfoliated graphene. Polym. Compos..

[53] Serban B.C., Buiu O., Dumbravescu N., Cobianu C., Avramescu V., Brezeanu M., Bumbac M., Pachiu C., Nicolescu C.M. (2021). Oxidized carbon nanohorn-hydrophilic polymer nanocomposite as the resistive sensing layer for relative humidity. Anal. Lett..

[54] Barkauskas J. (1997). Investigation of conductometric humidity sensors. Talanta.

[55] Yoo K.-P., Lim L.-T., Min N.-K., Lee M.J., Lee C.J., Park C.-W. (2010). Novel resistive-type humidity sensor based on multiwall carbon nanotube/polyimide composite films. Sensors Actuators B Chem..

[56] Caoa M., Wanga D., Lua J., Chenga W., Hana G., Zhoub J. (2021). Electrospun porous carbon nanofibers @ SnOx nanocomposites for high-performance supercapacitors: Microstructures and electrochemical properties. Compos. Part A Appl. Sci. Manuf..

[57] Ali F., Ishfaq N., Said A., Nawaz Z., Ali Z., Ali N., Afzal A., Bilal M. (2021). Fabrication, characterization, morphological and thermal investigations of functionalized multi-walled carbon nanotubes reinforced epoxy nanocomposites. Prog. Org. Coat..

[58] Elkodous M.A., El-Sayyad G.S., Maksoud M.A., Kumar R., Maegawa K., Kawamura G., Tan W.K., Matsuda A. (2020). Nanocomposite matrix conjugated with carbon nanomaterials for photocatalytic wastewater treatment. J. Hazard. Mater..

[59] Mansor M., Fadzullah S., Masripan N., Omar G., Akop M. (2019). Comparison between functionalized graphene and carbon nanotubes. Functionalized Graphene Nanocomposites and their Derivatives.

[60] Chatterjee A.P. (2006). A model for the elastic moduli of three-dimensional fiber networks and nanocomposites. J. Appl. Phys..

[61] Haghgoo M., Ansari R., Hassanzadeh-Aghdam M., Nankali M. (2019). Analytical formulation for electrical conductivity and percolation threshold of epoxy multiscale nanocomposites reinforced with chopped carbon fibers and wavy carbon nanotubes considering tunneling resistivity. Compos. Part A Appl. Sci. Manuf..

[62] Marinescu M.R., Serban B.C., Cobianu C., Dumbravescu N., Ionescu O., Buiu O., Ghiculescu L.D. (2021). Mechanical properties of carbon-based nanocomposites for sensors used in biomedical applications. UPB Bull. B Chem. Mater. Sci..

[63] Halphin J.C., Tsai S.W. (1969). Effect of Environment Factors on Composite Materials.

[64] Chen G.-X., Kim H.-S., Park B.H., Yoon J.-S. (2006). Multi-walled carbon nanotubes reinforced nylon 6 composites. Polymer.

[65] Kargarzadeh H., Mariano M., Huang J., Lin N., Ahmad I., Dufresne A., Thomas S. (2017). Recent developments on nanocellulose reinforced polymer nanocomposites: A review. Polymer.

[66] Kundalwal S.I. Review on Modeling of Mechanical and Thermal Properties of Nano- and Micro-Composites. https://arxiv.org/ftp/arxiv/papers/1708/1708.00764.pdf.

[67] Kumar D., Verma V., Bhatti H.S., Dharamvir K. (2011). Elastic moduli of carbon nanohorns. J. Nanomater..

[68] Treacy M.M.J., Ebbesen T.W., Gibson J.M. (1996). Exceptionally high Young’s modulus observed for individual carbon nanotubes. Nat. Cell Biol..

[69] Deng L., Eichhorn S.J., Kao C.-C., Young R.J. (2011). The effective young&rsquo;s modulus of carbon nanotubes in composites. ACS Appl. Mater. Interfaces.

[70] Dereli G., Sungu B. Temperature Dependence of the Tensile Properties of Single Walled Carbon Nanotubes; O(N) Tight Binding MD Simulation. https://arxiv.org/pdf/0704.0183.pdf.

[71] Yakobson B.I., Avouris P. (2001). Mechanical properties of carbon nanotubes. Advanced Structural Safety Studies.

[72] Healey J.N.C., Rubinstein M.H., Walters V. (1974). The mechanical properties of some binders used in tableting. J. Pharm. Pharmacol..

[73] Dodero E., Brunengo M., Castellano M., Vicini S. (2020). Investigation of the mechanical and dynamic-mechanical properties of electrospun polyvinylpyrrolidone membranes: A design of experiment approach. Polymers.

